# TUBER: Time-aware UAV-based energy-efficient reconfigurable routing scheme for smart wireless livestock sensor network

**DOI:** 10.1371/journal.pone.0292301

**Published:** 2024-01-05

**Authors:** Houssem R. E. H. Bouchekara, Abdulazeez F. Salami, Yusuf A. Sha’aban, Mouaaz Nahas, Mohammad S. Shahriar, Mohammed A. Alanezi

**Affiliations:** 1 Department of Electrical Engineering, University of Hafr Al Batin, Hafr Al Batin, Saudi Arabia; 2 Department of Computer Engineering, University of Ilorin, Ilorin, Nigeria; 3 Department of Electrical Engineering, Umm Al-Qura University, Makkah, Saudi Arabia; 4 Department of Computer Science and Engineering Technology, University of Hafr Al Batin, Hafr Al Batin, Saudi Arabia; University of Tabuk, SAUDI ARABIA

## Abstract

This paper is a follow-up to a recent work by the authors on recoverable UAV-based energy-efficient reconfigurable routing (RUBER) scheme for addressing sensor node and route failure issues in smart wireless livestock sensor networks. Time complexity and processing cost issues connected to the RUBER scheme are consequently treated in this article by proffering a time-aware UAV-based energy-efficient reconfigurable routing (TUBER) scheme. TUBER scheme employs a synchronized clustering-with-backup strategy, a minimum-hop neighborhood recovery mechanism, and a redundancy minimization technique. Comparative network performance of TUBER was investigated and evaluated with respect to RUBER and UAV-based energy-efficient reconfigurable routing (UBER) schemes. The metrics adopted for this comparative performance analysis are Cluster Survival Ratio (CSR), Network Stability (NST), Energy Dissipation Ratio (EDR), Network Coverage (COV), Packet Delivery Ratio (PDR), Fault Tolerance Index (FTI), Load Balancing Ratio (LBR), Routing Overhead (ROH), Average Routing Delay (ARD), Failure Detection Ratio (FDR), and Failure Recovery Ratio (FRR). With reference to best-obtained values, TUBER demonstrated improvements of 36.25%, 24.81%, 34.53%, 15.65%, 38.32%, 61.07%, 31.66%, 63.20%, 68.96%, 66.19%, and 78.63% over RUBER and UBER in terms of CSR, NST, EDR, COV, PDR, FTI, LBR, ROH, ARD, FDR, and FRR, respectively. These experimental results confirmed the relative effectiveness of TUBER against the compared routing schemes.

## 1. Introduction

Automated technologies and Wireless Sensor Network (WSN) innovations have been proffered by researchers as effective solutions to address the large-scale production challenges associated with livestock farming (LF) [1–10]. In addition to this, the integration of real-time smart computing into Internet of Things (IoT) systems for smart Sensor Nodes (SNs) have paved the way for efficient management of scarce energy resources [11, 12]. Therefore, optimal allocation and conservative consumption of scarce energy resources is cardinal to extending the lifespan of specialized WSNs employed for livestock monitoring [4, 6]. One of the conventional energy minimization approaches is to employ the use of low-cost small-sized, wearable, and smart SNs for observing, collecting, and processing livestock events [2, 11, 13]. Nonetheless, adverse weather conditions in conjunction with the erratic movement of livestock make tracking through wearable SNs very difficult and challenging [14–16]. These difficulties include poor network lifetime, rapid energy loss, erratic coverage for large monitoring field, high operational cost of concurrent tracking, and human measurement errors [17, 18].

These difficulties have resultantly motivated researchers on the effective adoption of Unmanned Aerial Vehicles (UAVs) to address sensing and routing issues associated with surveilling livestock events [3, 6, 7, 12, 14–17, 19, 20]. The core technological benefits of improving livestock farming (LF) surveillance with UAVs are: broader coverage, dynamic and seamless tracking, and better flexibility in network design [4, 21–26]. In such LF tracking scenarios, UAVs are employed as 1) sinks for collating prioritized events from strap-on SNs and 2) mobile hovering terminals for direct livestock inspection [27, 28]. By incorporating these functionalities into WSN solutions, researchers have designed and deployed a variety of integrated UAV-WSN solutions where UAVs are used as Mobile Sinks (MS) for collating livestock events, and customized smart computing algorithms are employed for data-driven decision making [9, 27]. Nevertheless, network lifetime truncation and coverage disruption issues still exist as open research problems.

Researchers have put forward a variety of traditional [29–36] and tailor-made [27, 37–46] routing approaches to tackle these problems. Among the propounded strategies, Cluster-Based Routing (CBR) schemes have demonstrated marked network performance improvements over flat-based, chain-based, and tree-based routing topologies for large-scale time-sensitive WSN applications [2, 47–49].

Implementation of a decentralized autonomous LF tracking using CBR approach requires scalable and tractable routing architecture. This architecture entails the systematic, logical and dynamic organization of livestock-attached SNs into groups, referred to as Herd Clusters (HCs) in the context of this article. In each HC, there is an elected HC Lead (HCL) with corresponding HC Members (HCMs). Primarily, the role of HCMs is to transmit livestock events to their corresponding HCLs using hop to hop delivery. HCLs thereafter forward aggregated data to the nearest MS for eventual transmission to the Base Station (BS) [2, 44, 46, 50, 51].

By relying on this herd CBR approach, this paper presents a time-aware UAV-based energy-efficient reconfigurable (TUBER) routing algorithm for smart wireless livestock sensor networks. This TUBER scheme is an improved version of the previous recoverable UAV-based energy-efficient recoverable routing (RUBER) and UAV-based energy-efficient recoverable routing (UBER) schemes proposed by the same authors. The major contributions of TUBER are:

➢ it addresses time-complexity and processing cost issues associated with the RUBER scheme,
➢ it also tackles SN and route failure issues faced by the UBER scheme.
➢ it relies on a synchronized clustering-with-backup strategy, minimum-hop neighborhood recovery mechanism, and redundancy minimization technique

These algorithmic techniques and operational mechanisms introduced for TUBER are functional improvements, specialized enhancements, and algorithmic extensions over the operational features of RUBER and UBER. OMNET++ and MATLAB experiments were extensively conducted for comparative performance analysis with respect to RUBER and UBER schemes. Experimental results confirmed the relative effectiveness of TUBER against the compared routing schemes.

The remainder of this paper is organized as follows: Section 2 surveys relevant MS-based fault-tolerant CBR schemes. Section 3 provides an operational description for the proposed TUBER scheme, while Sections 4 and 5 present the obtained experimental results and pertinent technical discussions/justifications, respectively. The conclusion of this paper and future research works are delivered in Section 6 and Section 7, respectively.

## 2. Related work

Most of the common CBR techniques are normally classified as generic based on their flexible mode of operation and relevance to a variety of WSN applications. Notwithstanding, traditional CBR techniques are usually plagued with challenges such as time-complexity trade-off, processing costs, recurrent HCL failures, rapid energy loss, network re-sizing/re-scaling issues, and cluster re-configuration problems [29–36, 47, 48, 52–54].

RUBER was propounded as an energy-efficient, fault-tolerant and recoverable CBR scheme to address sensor node and route failure issues in smart wireless livestock networks [55]. This CBR scheme employs resilient route maintenance strategy, effective recovery mechanism, and robust fault detection and recycling methodology. Time-varying load balancing parameter was formulated, monitored and finetuned for effectively regulating HC size, equalizing traffic load, and curbing network congestion [55]. The limitations of this CBR scheme are the time complexity and processing cost issues.

UBER was proposed as a UAV-based energy-efficient, and reconfigurable CBR technique to tackle coverage loss and energy dissipation issues for smart wireless livestock sensor networks [2]. This CBR technique relies on dynamic energy thresholding strategy, UAV-aided coverage recovery technique, and adjustive inter-cluster route formation. HCL election criteria are distance (from HCL to aerial MS) and cluster formation costs while hop-by-hop delivery is employed for end-to-end communication in this specialized WSN application [2]. The drawbacks of this CBR technique are the fault-tolerant challenges associated with SN and route failures.

FCGW was proffered as a resilient CBR scheme that adopts Gaussian network configuration, transmission path alignment, multi-path link formation, and grid-based cluster construction [37]. The LF network space is partitioned into logical grids (square-shaped clusters) while HCL election is conducted with the aid of Gaussian integers. Network re-sizing and load balancing is managed with the assistance of aerial MS. Multi-path transmission coupled with computation of minimal inter-cluster distance costs are employed for end-to-end communication in the custom-made Gaussian WSN [37]. The deficiencies of this CBR scheme are high end-to-end delays and control overhead issues incurred for long-distance communication in the proposed Gaussian network.

HYBRID was offered as a lifespan enhancement CBR technique by utilizing SNs dispersed in harsh LF environments to achieve energy-efficiency through logical partitioning of the LF field into HCs and positioning the BS at the edge of the field [38]. HC configuration and HCL election probability are done based on residual energy while cluster distance computations are employed for multi-hop communication of livestock events in the LF network [38]. Processing costs of implementing multi-transmission modes and multiple energy settings are the shortcomings of this CBR strategy.

MS-DVCR was proffered as an energy-minimization geographic routing approach that utilizes MS-assisted target-oriented virtual coordinate data transmission in the network [39]. Virtual coordinate transmission technique helps to reduce data aggregation control packets and updates during to MS-to-BS communication. Through this geographic routing approach, alternative MS localization is effectively achieved without the need for physical distance measurements [39]. The defects of this routing approach are the runtime overhead resulting from processing, maintaining, managing and broadcasting virtual coordinate packets.

AODV-SMS (ABC-PSO) was put forward as a fault-tolerant route-optimization CBR strategy that relies on particle swarm and artificial bee colony algorithms for mobile WSN applications [27]. The methodology of this CBR strategy adopts alternative route optimization, particle selection, collaborative updating, path coding, and swarm evolution in order to achieve transmission path reliability, computational accuracy, rapid recovery, and network parameter optimization [27]. The major deficiencies of this CBR strategy are the energy tax and computational overhead for optimizing the network parameters.

VBRP was proposed as a connectivity-aware delay-minimization CBR algorithm that employs rendezvous points computation, hop-by-hop delivery, orphaned SN re-affiliation, transmission chain reduction, and Voronoi vertices for MS route optimization in critical WSN applications [40]. By implementing this CBR algorithm, network adaptability, reduced transmission delay, optimal MS route computations, and prolonged network lifetime are achieved in the network [40]. The drawbacks of this CBR algorithm are frequent coverage outage, energy loss, and poor load balancing.

RRM-WLDCNN was proffered as a fault-tolerant routing strategy that adopts deep learning-based (convolutional neural network) route prediction, subgraph extraction, and graph labelling for ensuring high reliability in specialized WSN monitoring applications [41]. The advantages of this routing strategy are energy conservation, reduced transmission cost, improved network lifespan, and enhanced route reliability [41]. The limitations of this routing strategy are processing overheads, coverage interruptions, scalability issues, and non-adaptability to heterogeneous WSN applications.

Table 1 gives the summary and highlights the key technical differences of the CBR schemes reviewed in the related works. The novelty of the TUBER scheme with respect to these reviewed CBR schemes is the synchronized clustering-with-backup strategy, minimum-hop neighborhood recovery mechanism, and redundancy minimization technique introduced in this research work.

**Table 1:** Summary of reviewed CBR schemes [55].

CBR Scheme	Authors	Year	Taxonomy of CBR Scheme	Mechanism	Metrics	Benefits	Drawbacks
RUBER	Bouchekara et al.	2022	Energy-minimization, fault-tolerant and recoverable	Route maintenance strategy, failure recovery mechanism, fault detection/ recycling mechanism	Failure detection ratio, failure recovery ratio, load balancing ratio, and packet delivery ratio, average routing delay, energy dissipation ratio, routing overhead, fault tolerance index, nodal failure recovered, route failure recovered, and cluster survival ratio	Fault-tolerance, network resilience	Time-complexity and processing cost issues
UBER	Alanezi et al.	2022	Energy-minimization and reconfigurable	Dynamic residual energy thresholding, cluster-to-UAV link formation, UAV-assisted coverage recovery	Network stability, load balancing ratio, topology fluctuation effect ratio, energy consumption, network coverage, received packets, SN failures detected, route failures detected, routing overhead, end-to-end delay	Energy conservation, network coverage maximization	Critical SN and route failures
FCGW	Quoc et al.	2022	Fault-tolerant	Multi-path routing, symmetric link formation, Gaussian network architecture, square-shaped cluster formation	Average residual energy, number of dead nodes, number of packets sent/received	Enhanced fault tolerance, improved data reliability, minimal energy consumption	Broadcast overhead issues, large delays from long-range transmissions
HYBRID	Behera et al.	2019	Network lifetime enhancement	Distance-dependent hybrid routing mode, two-mode energy level for effective power utilization	Residual energy, packets received, throughput, number of alive nodes, number of dead nodes, network stability, network lifetime	Improved throughput, prolonged network lifetime, minimal energy consumption	Processing costs of operating varying transmission modes and energy levels
MS-DVCR	Rahmatizadeh et al.	2014	Energy-minimization and geographic	MS-based goal-oriented virtual coordinate technique	Successfully routed messages, average path length, energy consumption	Lower energy consumption, effective mobile nodes localization	Execution overhead from virtual coordinate information broadcast, maintenance, and management
AODV-SMS	Yue et al.	2018	Route-optimization and fault-tolerant	Artificial bee colony-particle swarm algorithm	Energy consumption, energy utilization rate, packet loss rate, transmission latency, network connectivity, network reliability, size of network	Reliable data transmission route, effective route recovery ability, accurate computations of network parameter(s) optimization	Computational cost for running the optimization routine, high energy tax
VBRP	Nitesh et al.	2017	Delay-minimization and connectivity-aware	Local orphaned SN recovery strategy, single-hop communication, Voronoi vertices	Path length, average waiting time, network lifetime, standard deviation of remaining energy	Extended network lifespan, optimal MS path length, reduced average waiting duration, better network adaptability	Poor energy balancing, coverage loss issues from topology variations
RRM-WLDCNN	Huang et al.	2020	Fault-tolerant and deep learning-based	Graph labelling, subgraph extraction, path prediction using deep-learning (kernel and dual convolutional neural network)	Transmission delay, system residual energy, average routing length, number of dead nodes, precision	Lower data transmission cost, improved path reliability, enhanced network lifetime, better energy consumption	Interference issues, processing delays, lack of flexibility/adaptability

## 3. Proposed TUBER scheme

This section discusses the integrated system model, synchronized clustering-with-backup process, data gathering and forwarding procedure, minimum-hop neighborhood recovery mechanism, and redundancy minimization technique.

### 3.1. Integrated system model

**Fig 1:**
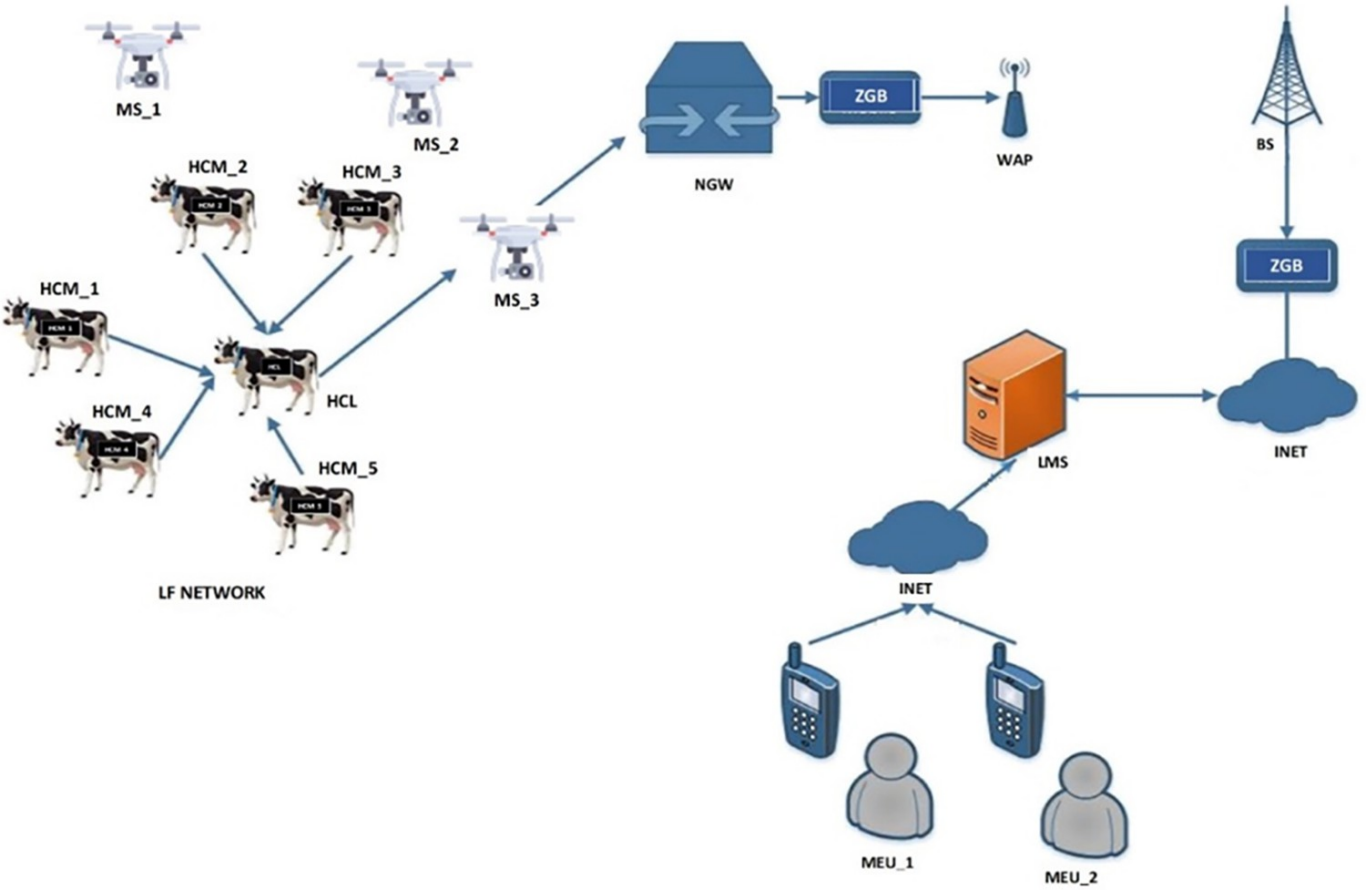
Integrated system model [2].

Fig 1 describes the integrated system model with a heterogeneous network arrangement for smart wireless livestock sensor network. The system architecture depicts the flow of information from the moment livestock events (heart rate, sweat concentration, temperature, location) are detected in the representative HC and eventually transmitted to the desired destination. With reference to the diagram, NGW, ZGB, LMS, WAP, INET, MEU, BS, HCM, HCL, LFC, and MS represent network gateway, ZigBee interface, livestock monitoring server, wireless access point, internet link, mobile end user, terrestrial base station, herd cluster member, herd cluster lead, livestock farming controller devices (auto-switchers, heat regulators, alarms, lightning controllers, and other actuators), and aerial mobile station, respectively.

The functional and electronic properties of the UAVs suitable for the integrated model presented in Fig 1 are subsequently explained with elaborate details in [55]. The UAVs have more onboard radios to accommodate the influx of periodic traffic from different clusters and they are deployed to follow the livestock herd within the LF network perimeter. The movement profile and scanning pattern of the UAV is in x, y, z direction following straight-line left-to-right up-and-down scanning pattern with specific spatial mobility limitations (no axial rotation, no angular twists/bends). Velocity profiles for the UAV are stationary, scanning velocity (20 m/s) as stated in Table 3, and data gathering velocity (5 m/s). The cognitive capability of the UAV is not fully autonomous as its activities can be controlled from the LMS. The reason for utilizing this semi-autonomous arrangement is to prevent out-of-perimeter straying which can lead to lost UAV, theft, unrestrained energy loss or device damage. The UAV’s normal altitude is operated at 230m which falls within the SN’s transmission range of 250m in order to preserve network coverage and connectivity. Command and control signaling is used to enable sending of command signal to UAV from the LMS and receiving data traffic from UAV payload [55]. Readers are referred to [2, 55] for further details on the network flow, operations, underlying assumptions and energy consumption model pertinent to this integrated system model.

### 3.2. Synchronized clustering-with-backup process

The goal of the synchronized clustering-with-backup strategy is to ensure that HCL election is simultaneously executed with backup route selection. TUBER algorithm starts with network initialization by computing and exchanging clustering costs (*CC*_x_) among adjacent SNs. The set of adjacent SNs (*G*_ADJ_) is constructed while the identity of the final HCL and backup routes are kept as NULL. *CC*_x_ is computed as [2]:

$${CC}_{x}=max[Z_{x,t}]$$ (1)

Where *Z*_x,t_ is the signal strength of UAV beacon received by SN_x_ at period *t*. After the initialization stage, SNs with established link with MS contend to take up HCL roles through computation and dynamic update of their electability probability (*HCL*_PR_) as:

$${HCL}_{PR}=\left\{ \begin{array}{l} max[\frac{E_{rsd}}{\left({LIM}_{UP}\times E_{cor}\right)},{LIM}_{LOW}],if\;{CC}_{x}>0\\ 0,\;if\;{CC}_{x}=0 \end{array}\right.$$ (2)

Eq (2) is periodically monitored, updated and regulated with a bounded condition as:

$$[{LIM}_{UP}\times E_{cor}]>E_{tot}$$ (3)

Where *E*_tot_, *E*_rsd_, *E*_cor_, *LIM*_UP_ and *LIM*_LOW_ are total energy, residual energy, energy consumption rate, upper bound for HCL contention, and lower bound for HCL contention. The *E*_rsd_/*E*_tot_ ratio introduced in [2, 55] is more suitable for a scenario where all SNs commence (and operate) with equal energy resources but this premise does not totally conform with the heterogeneous network model where energy resources are usually not equally distributed due to varying energy consumption rate at different network rounds. A more lifetime-sensitive ratio of *E*_rsd_/*E*_cor_ is therefore introduced in Eq (2) for TUBER to cater for heterogeneous battery consumption trends. *E*_cor_ captures energy consumed to carry out extra-network functions such as scheduling, processing, amplification, fusion, and media access control.

**Fig 2:**
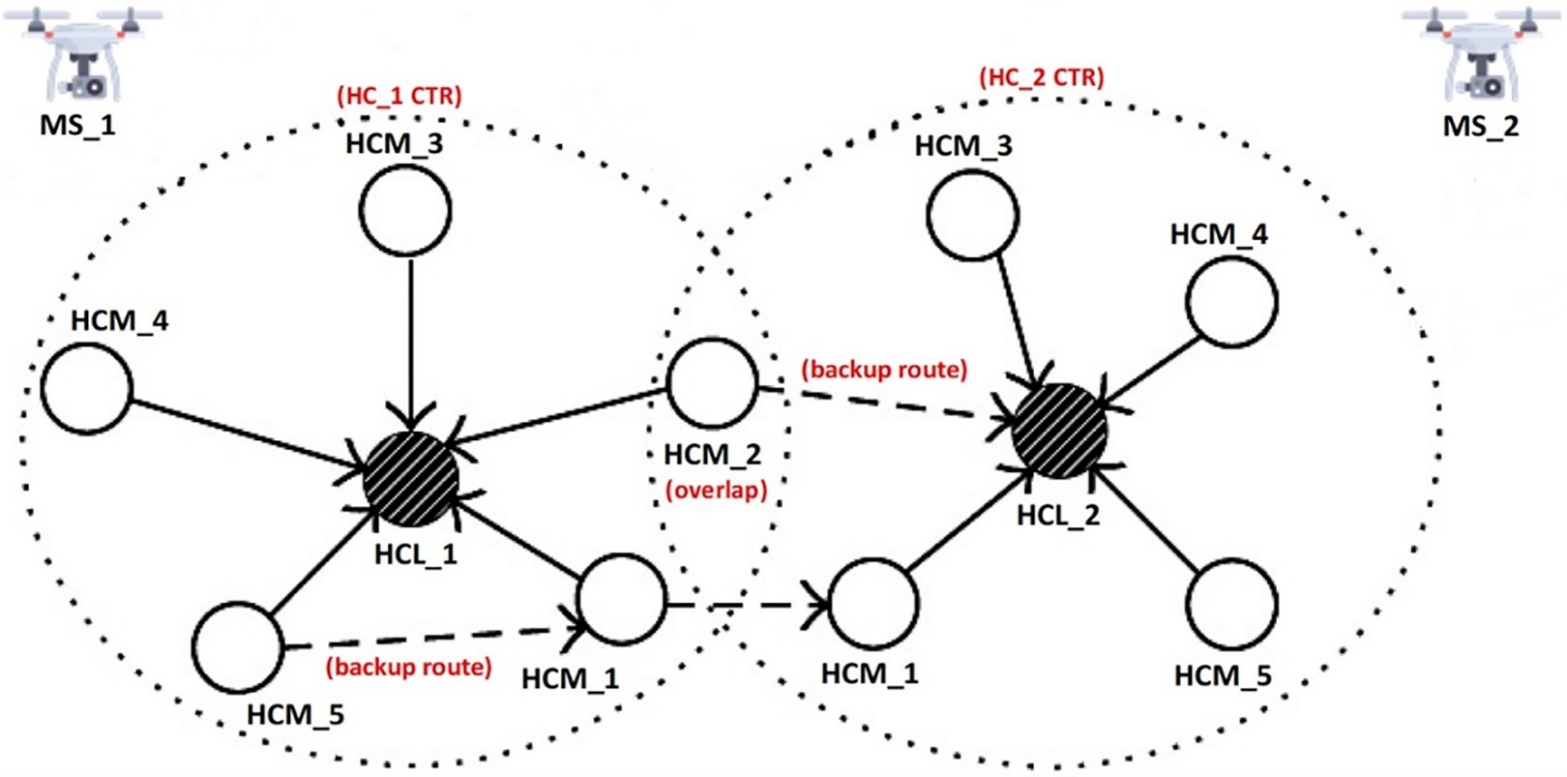
Synchronized clustering-with-backup process.

In the contention phase, the set of neighboring trial HCLs (*G*_THCL_) is constructed based on contiguous SNs with highest *HCL*_PR_ values and least *CC*_x_. It must be mentioned that *CC*_x_ acts as a tiebreaker when there are multiple SNs returning similar *HCL*_PR_ values. Furthermore, *G*_THCL_ information is broadcasted with nearest SNs and the trial HCLs with the least *CC*_x_ are selected, upgraded (role-wise) and confirmed as final HCLs. The backup route selection is synchronized and simultaneously carried out with the clustering process as shown in Fig 2. For example, HCM_2 learns through network discovery that the direct route to HCL_2 is stored for backup. On the other hand, HCM_1 of HC_1 cannot directly reach HCL_2 and as a result, HCM_1 of HC_2 is kept as a backup route to HCL_2. In the same fashion, HCM_5 of HC_1 adopts the backup route of HCM_1 of HC_1 to establish backup route to HCL_2. This ensures that least-cost multi-hop backup transmission chains are constructed in this manner.

During the HCM enrolment process, final HCLs broadcast POLLING packets to their adjacent SNs while the receiving SNs respond with JOIN packets accordingly. The HCM enrolment process concludes with fully learning of the backup routes and allocating HCMs to their respective final HCL. The synchronized clustering-with-backup algorithm is described in Table 2 while the pictorial description of this process is given in Fig 2. The resulting flowchart for the synchronized clustering-with-Backup algorithm is also presented in Fig 4.

**Table 2:** Synchronized clustering-with-backup algorithm for TUBER.

**Input:** Z_x,t_, E_tot_, E_rsd_, E_cor_, LIM_UP_, LIM_LOW_ **Output:** final_HCL, BCUPRUT, CC_x_, HCL_PR_ 1: **for** each SN_x_ {rcv} MS_CONNECT beacon
2: **if** (Z_x,t_ ← MS && Z_x,t_ ≠ NULL)
3: status.connect(MS) ← TRUE
4: compute CC_x_ ← find_peak(Z_x,t_)
5: **else**
6: status.connect(MS) ← FALSE
7: CC_x_ ← -INF
8: **end if**
9: broadcast CC_x_ → SN_x_.ADJ ∈ CTR
10: status.final_HCL ← FALSE
11: status.BCUPRUT ← FALSE
12: **end for**
13: **while** (R ≠ R_max_ && HCL_PR_ ≠ 1)
14: **if** (status.connect(MS) ← TRUE && G_THCL_ ≠ ∅)
15: HCL_PR_ ← rand(0,1)
16: elect.MY_HCL ← trial_HCL.min(CC_x_)
17: status.BCUPRUT ← TRUE
18: BCUPRUT_EDGE ← Euclid(CC_x_, SN_x_.ADJ, 2)
19: **if** (MY_HCL = SN_ID && HCL_PR_ ≠ 1)
20: broadcast HCL_polling(SN_ID, trial_HCL, CC_x_)
21: status.final_HCL ← FALSE
22: update(status.BCUPRUT)
23: **end if**
24: **else**
25: broadcast HCL_polling(SN_ID, final_HCL, CC_x_)
26: update(status.BCUPRUT)
27: **end if**
28: HCL_PR_ at t-1 ← HCL_PR_
29: HCL_PR_ ← min(2xHCL_PR_,1)
30: check bound(E_tot_, E_cor_, LIM_UP_)
31: **end while**
32: status.final_HCL ← TRUE
33: status.BCUPRUT ← TRUE
34: update(trial_HCL) ← POLLING
35: update(status.BCUPRUT)
36: elect(trial_HCL) ← IDLE
37: elect(final_HCL) ← ACTIVE
38: **if** (MY_HCL ≠ SN_ID && |G_THCL_| > 1)
39: BCUPRUT_EDGE ← Euclid(CC_x_, SN_x_.ADJ, 2)
40: **else** BCUPRUT_EDGE ← register(SNx.ADJ, final_HCL ≠ MY_HCL, ACTIVE)
41: **end if**
42: **if** (status.BCUPRUT ← FALSE)
43: BCUPRUT_EDGE ← register(SNx.ADJ, BCUPRUT_EDGE ≠ MY_HCL_EDGE, ACTIVE)
44: **else** BCUPRUT_EDGE ← register(SN_ID, MY_HCL, ACTIVE)
45: **end if**
46: broadcast POLLING(SN_ID, MY_HCL, BCUPRUT_EDGE, ACTIVE) ∈ CTR
47: **for** each SN_x_ {rcv} POLLING
48: Euclid(CC_x_, SN_x_.ADJ, 1)
49: multicast JOIN packet
50: update(status.BCUPRUT)
51: **end for**
52: **if** SN_x_! {rcv} POLLING
53: Euclid(CC_x_, SN_x_.ADJ, 1) ∈ CTR
54: MAIN_EDGE ← least Euclid() return
55: update(status.BCUPRUT)
56: **end if**
57: **for** each HCL
58: register HCM list
59: construct MAIN_EDGE ⊆ HCM
60: store updated BCUPRUT_EDGE set
61: **end for**

### 3.3. Data gathering and forwarding procedure

In this steady-state stage, HCMs are triggered to ON state for detecting and transmitting livestock events to their respective HCLs. To reduce processing cost, TUBER’s steady-state process uses only Neighborhood-1 HCMs with HCL-based joint handshaking. This initiative is an improvement over RUBER which employs Neighborhood-1 HCMs, Neighborhood-2 HCMs, and repeated separate (SN-based) handshaking [55]. Fig 2 illustrates the event detection and transmission process for TUBER. As an illustration, Neighborhood-1 HCMs of HC_1 (HCM_5, HCM_1, HCM_4, HCM_3, HCM_2) detects livestock events peculiar to their targets and forward the desired events to HCL_1. Upon successful packet delivery and provided there are no SN or route failures, HCL_1 handshakes with each Neighborhood-1 HCMs through ACK packets. HCL_1 aggregate all received events and forward the accumulated event to MS_1. Overlap HCMs (e.g., HCM_2) are not considered as cluster classification error but used strategically for backup route selection. Upon successful delivery of the required aggregated events to the LMS by MS_1 and MS_2 after *R*_max_ (maximum operation round), INTERRUPT beacon is signaled to the HCLs for the END_ROUND session.

### 3.4. Minimum-hop neighborhood recovery mechanism

**Fig 3:**
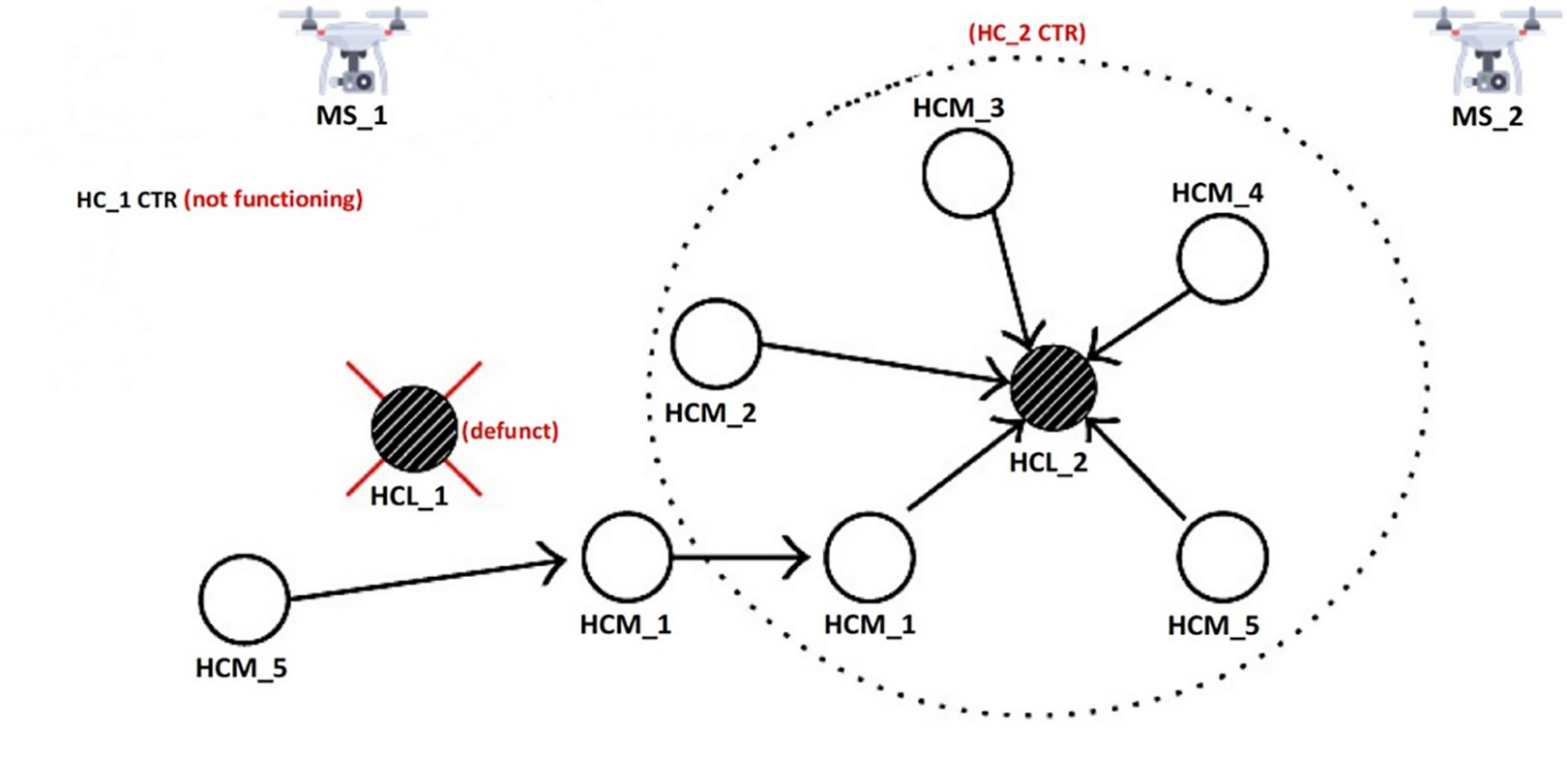
Minimum-hop neighborhood recovery mechanism.

Fig 3 depicts a scenario where HC_1 is not functioning and HCL_1 is classified as defunct (i.e., dead receiving, transmitting, sensing, microcontroller, and power modules) based on the recycling strategy discussed in [55]. In this case, the updated backup route (learned synchronously during clustering process) is triggered and employed. For this instance of SN failure of HCL_1 (defunct category), HCM_5 of HC_1 employs the shortest backup route (using second least CCx hop cost) to HCM_1 of HC_1. HCM_1 of HC_1 forwards the detected event to HCM_1 of HC_2. HCM_1 of HC_2 forwards the detected event to HCL_2. After handshaking with Neighborhood-1 HCMs, HCL_2 conducts pre-processing, aggregating, and forwarding of collated events to MS_2 for eventual delivery to the LMS. The minimum-hop neighborhood recovery mechanism is presented in Fig 3.

### 3.5. Redundancy minimization mechanism

**Fig 4:**
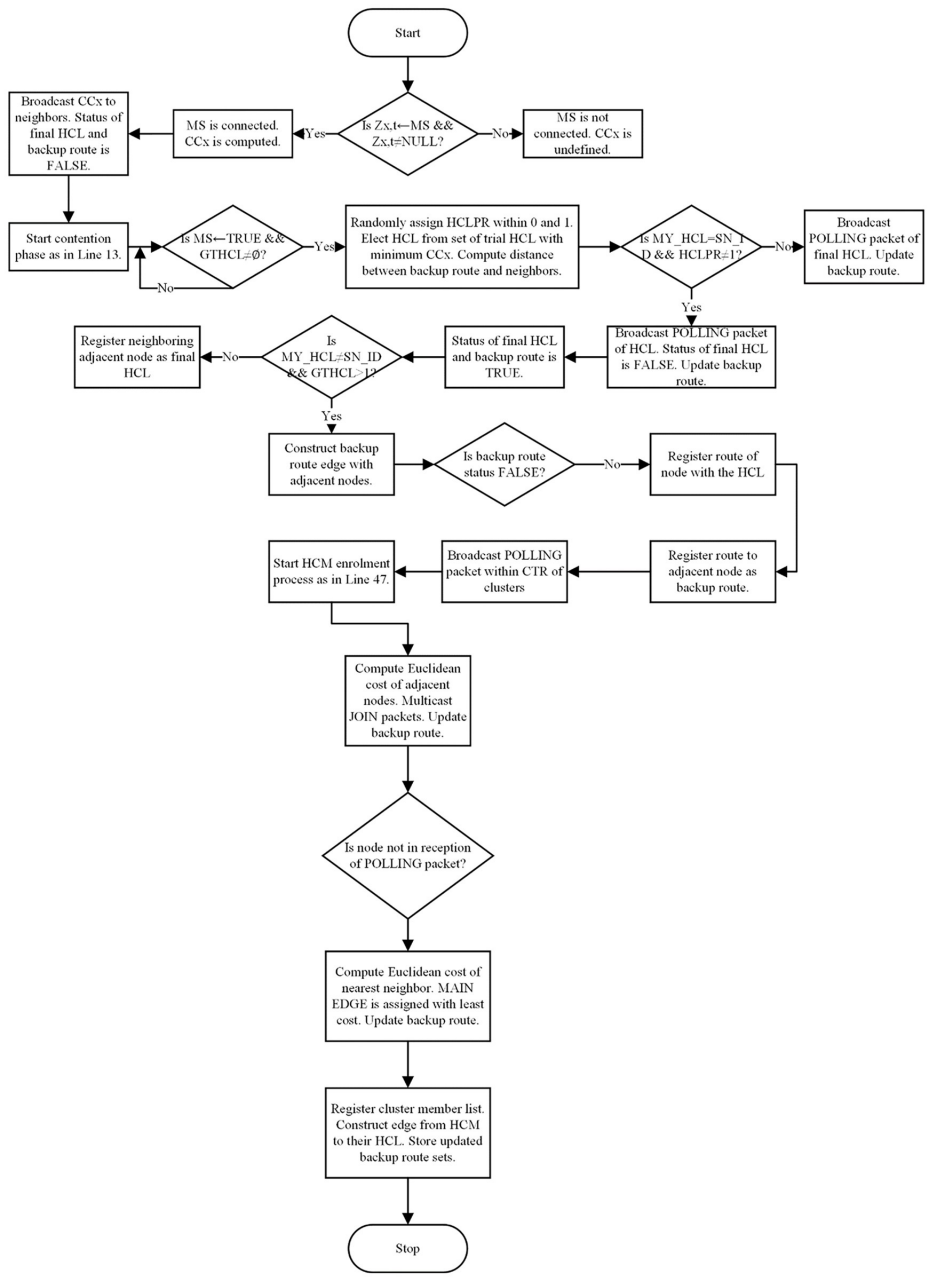
Flowchart of synchronized clustering-with-backup algorithm.

The function of the redundancy minimization strategy is to 1.) ensure fault-tolerance and network survivability without the deployment of redundant sensors or special privileged HCLs as backups, 2.) prevent recurrent full re-clustering after detecting SN and route failures, and 3.) minimize redundancy caused by idle backups associated with over-reliance on SN density. Specifically, the synchronized clustering-with-backup implemented in TUBER helps to avoid recurrent full re-clustering and idle backups–kindly refer to Fig 4. This is because TUBER utilizes partial re-clustering (rapid re-affiliation to contiguous HCs through previously learned backup routes) upon the detection of SN and route failures. Partial re-clustering leads to lesser energy consumption and processing costs in comparison to recurrent full re-clustering. Moreover, the fault-tolerance strategy for TUBER is decentralized (i.e., recovery is tailored to each SN) and not centralized (i.e., recovery is tailored to each cluster). The strength of TUBER’s decentralized fault-tolerant approach is to guarantee reliability and ensure the fault-tolerant function does not fail if the HCL fails or HC is not functioning.

## 4. Experimental results

This section is a technical exposition of TUBER’s performance metrics, experimental/simulation setup, time complexity, comparative performance results of TUBER in comparison with RUBER and UBER.

### 4.1. Performance metrics

The metrics adopted for comparative performance analysis are Cluster Survival Ratio (CSR), Network Stability (NST), Energy Dissipation Ratio (EDR), Network Coverage (COV), Packet Delivery Ratio (PDR), Fault Tolerance Index (FTI), Load Balancing Ratio (LBR), Routing Overhead (ROH), Average Routing Delay (ARD), Failure Detection Ratio (FDR), and Failure Recovery Ratio (FRR).

### 4.2. Simulation setup

Experiment setup through simulations was implemented with OMNET++ and MATLAB while setup parameters employed for the experiment are provided in Table 3. Other relevant network design specifications are based on the settings described in [2].

**Table 3:** Setup parameters.

Symbol	Description	Value
SN-DEP	Deployed SNs	250
LF-NS	Network Dimension	2000m x 2000m
PKS	Size of Data Packet	500 bytes
HELLO-PKS	Size of HELLO Control Packet	65 bytes
ACK-PKS	Size of ACK Control Packet	20 bytes
*E*_TAX_ TL	Energy Threshold Levels	8
SS-LR	Sensor-to-Sensor Link Rate	250 kbps
SMS-LR	Sensor-to-UAV Link Rate	1.75 Mbps
*E*_tot_	Total Initial Energy	2J
*E*_idle_	Idling Energy	0.2μJ
*E*_agg_	Collation Energy	5pJ/bit
*E*_EC_	Electronic Energy	5nJ/bit
*CTR*_max_	Maximum Coverage	250m
*Α*	Path Loss Factor	2.5
*SN-RS*	Sensitivity of Receiver	-95 dBm
*MS-ALT*	UAV Altitude	230m
*MS-V*	UAV Speed	20 m/s
*MS-SR*	UAV Beaconing Rate	2s
*MS-TD*	UAV Tour Length	960s
*AVG-STAT*	Statistical Averaging Runs	50

### 4.3. Time complexity

For the proposed TUBER scheme, the while loop (Line 13–31 of the algorithm) constitutes the most critical time delay component in the algorithm. By inspection, it follows that if this time delay component iterates *k* times before terminating at HCL_PR_ ≥ 1 with HCL_PR_ = LIM_LOW_ and doubled for each iteration (worst-case scenario), then the duration of the time delay lasts for:

$${LIM}_{LOW}\times 2^{k-1}<1$$ (4)

$$\overset{yields}{\to }k<1+\log_{2}\frac{1}{{LIM}_{LOW}}$$ (5)

$$\overset{yields}{\to }k\leq \left\lceil \log_{2}\frac{1}{{LIM}_{LOW}}\right\rceil$$ (6)

From Eq (6), LIM_LOW_ is a constant in the algorithm and as a result, TUBER has time complexity of *Θ*(1) which is relatively lower than the complexity (*Θ*(N)) of RUBER. With respect to the processing cost, if each broadcast operation (Line 9, 20, 25 & 46 of the algorithm) sends one packet for instance, then the worst-case analysis of the processing cost complexity also yields a constant as:

$$4+\left\lceil \log_{2}\frac{1}{{LIM}_{LOW}}\right\rceil$$ (7)

### 4.4. Performance evaluation

**Fig 5:**
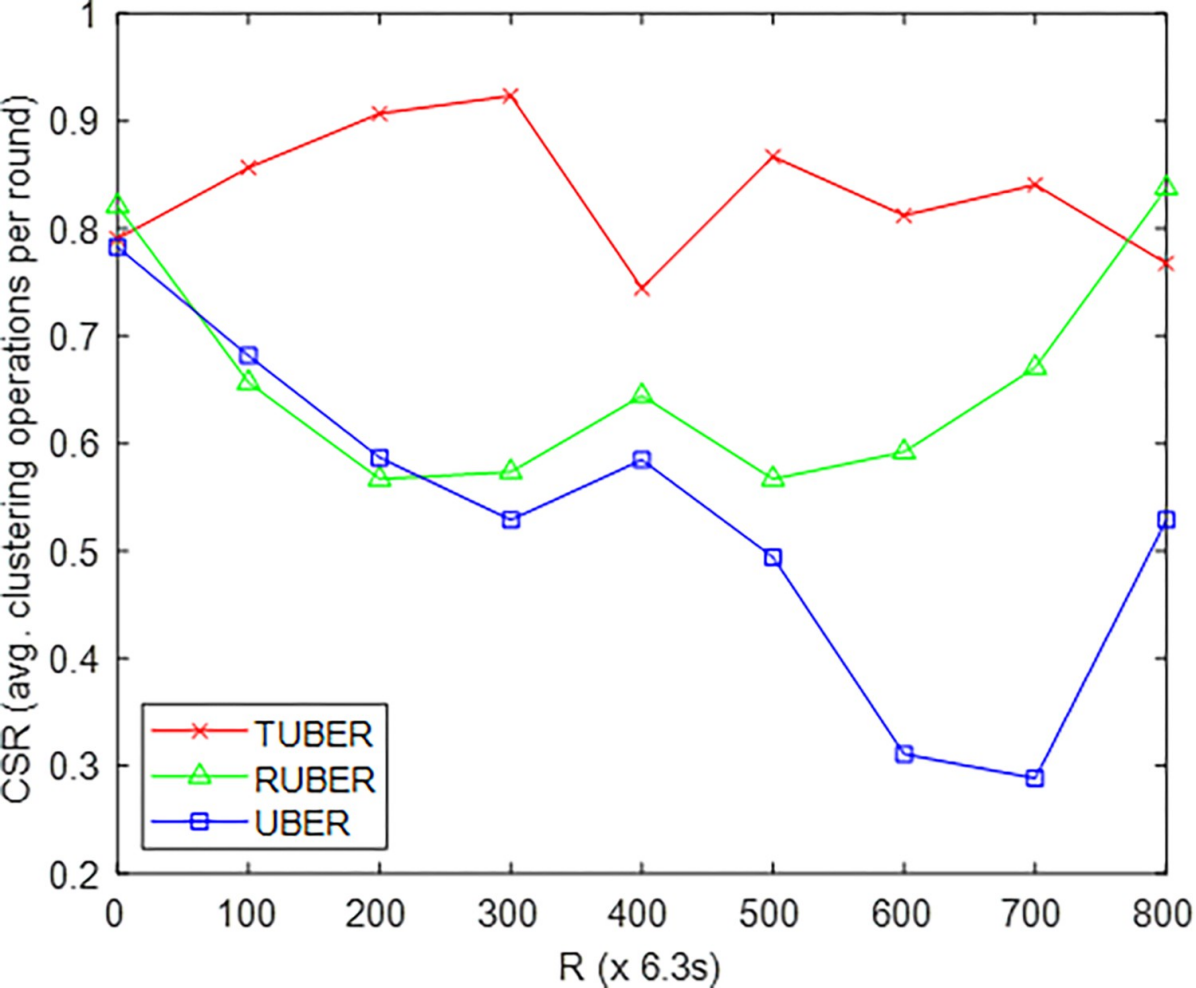
Cluster survival ratio performance.

In order to conduct comparative performance assessment, RUBER and UBER routing schemes were selected for benchmarking against TUBER. Fig 5 provides the comparative plot of cluster survival ratio (CSR) for TUBER against RUBER and UBER. CSR is defined as the ratio of average duration recorded for active clustering operations to the total network duration for each specific round.

**Fig 6:**
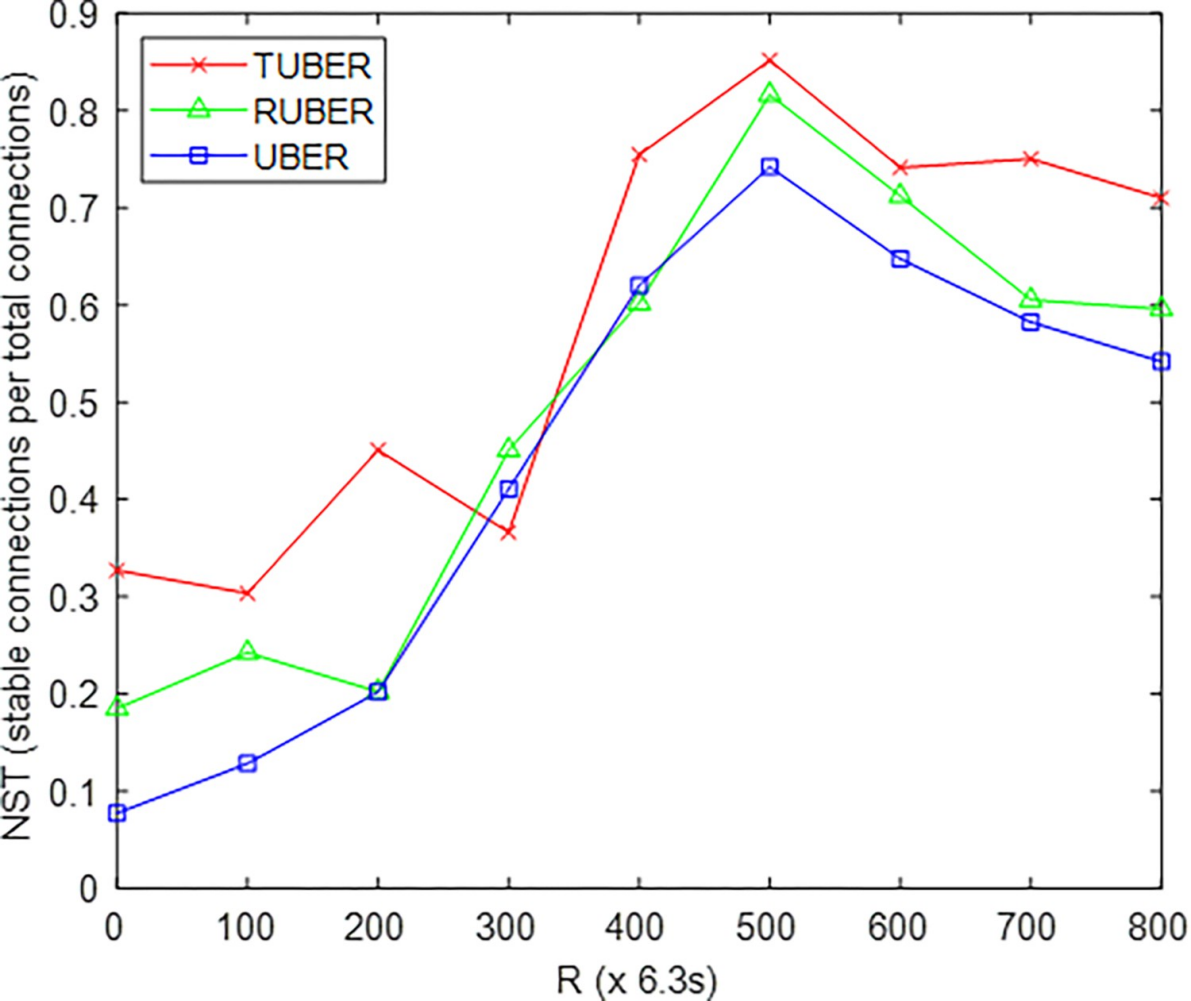
Network stability performance.

Fig 6 shows the comparative plot of network stability (NST) for TUBER with respect to RUBER and UBER. NST is defined as the ratio of the number of stable HCL-to-MS connections to the total number of connections after *R* network operation rounds.

**Fig 7:**
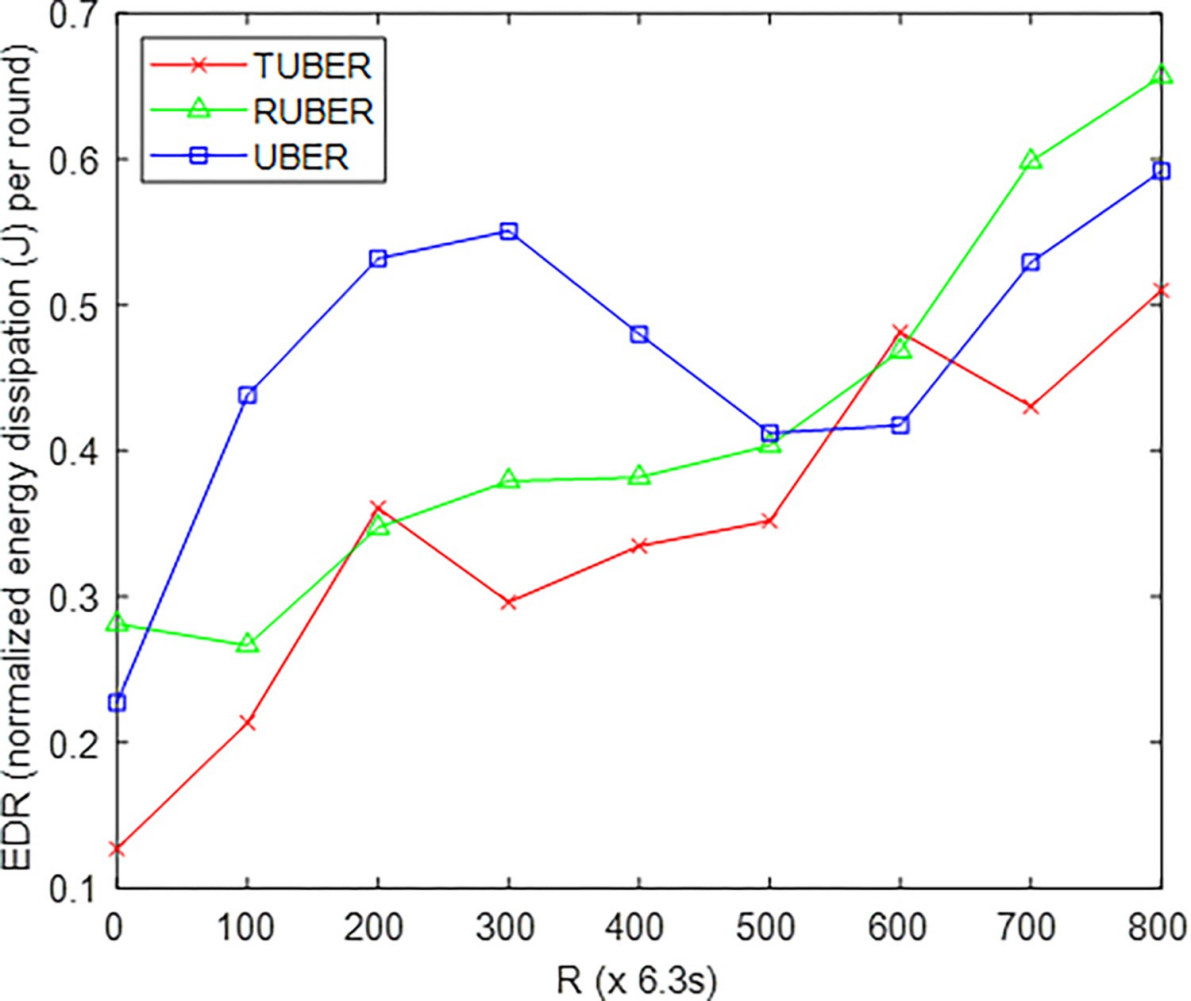
Energy dissipation ratio performance.

Fig 7 provides the comparative plot of energy dissipation ratio (EDR) for TUBER compared to RUBER and UBER. EDR is defined as the ratio of aggregate energy tax (E_TAX_) by the SNs to the total energy (E_tot_) after *R* network operation rounds.

**Fig 8:**
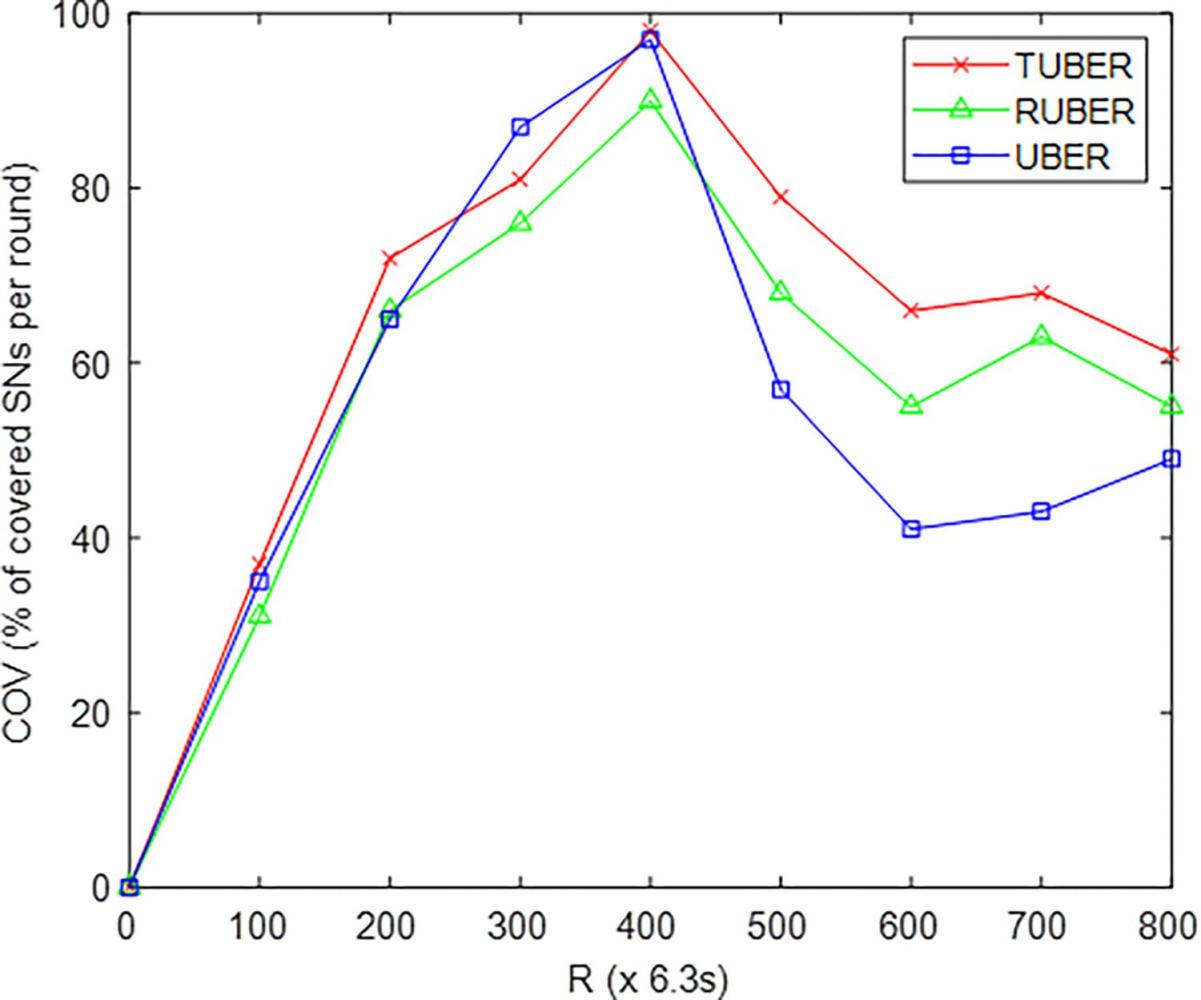
Network coverage performance.

Fig 8 depicts the comparative plot of network coverage (COV) for TUBER with respect to RUBER and UBER. COV is defined as the percentage of successfully covered SNs to the total node density for the LF network field.

**Fig 9:**
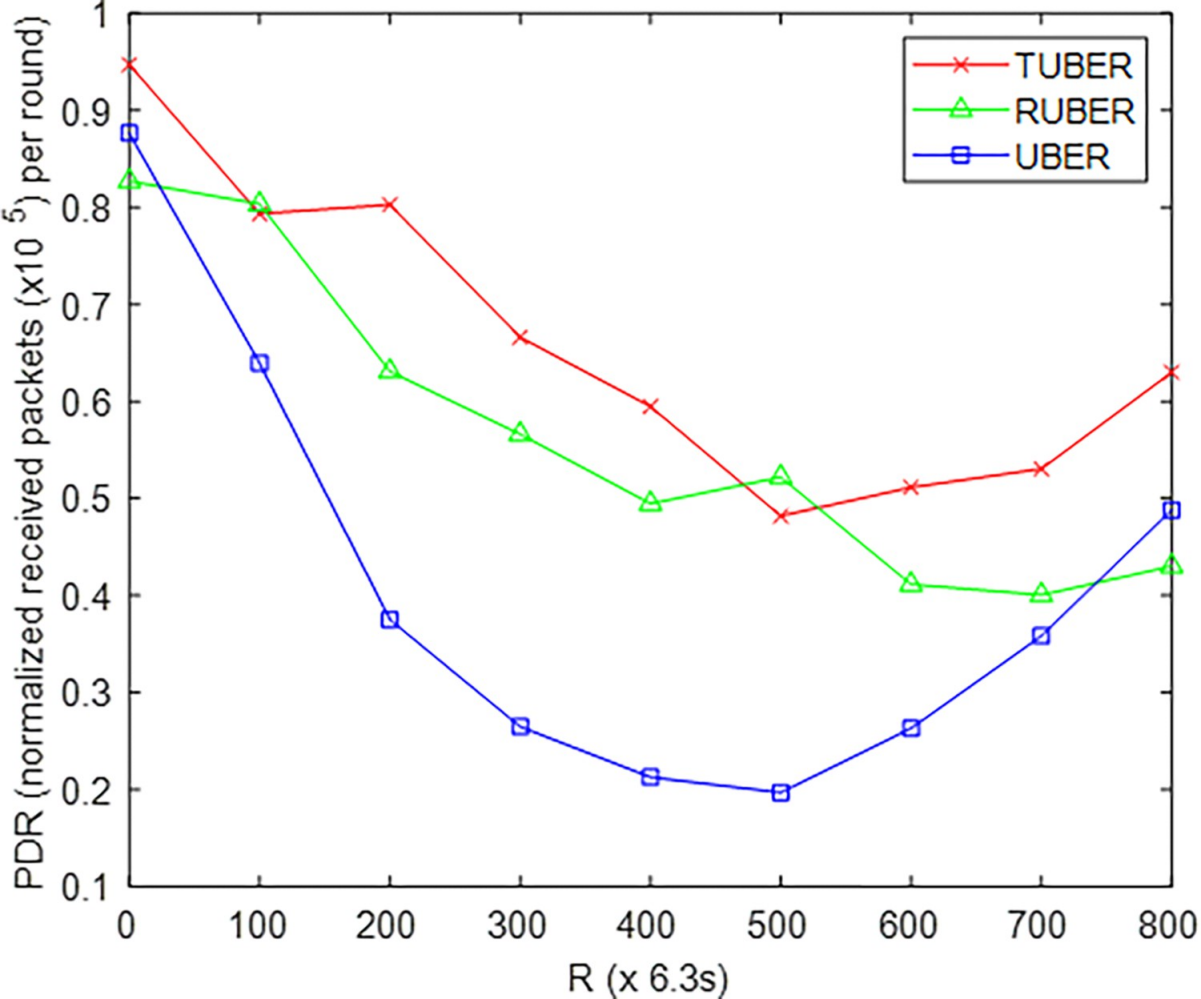
Packet delivery ratio performance.

Fig 9 shows the comparative plot of packet delivery ratio (PDR) for TUBER against RUBER and UBER. PDR is defined as the ratio of total received packets recorded via the MS-to-LMS transmission link to the total number of packets sent after *R* network operation rounds.

**Fig 10:**
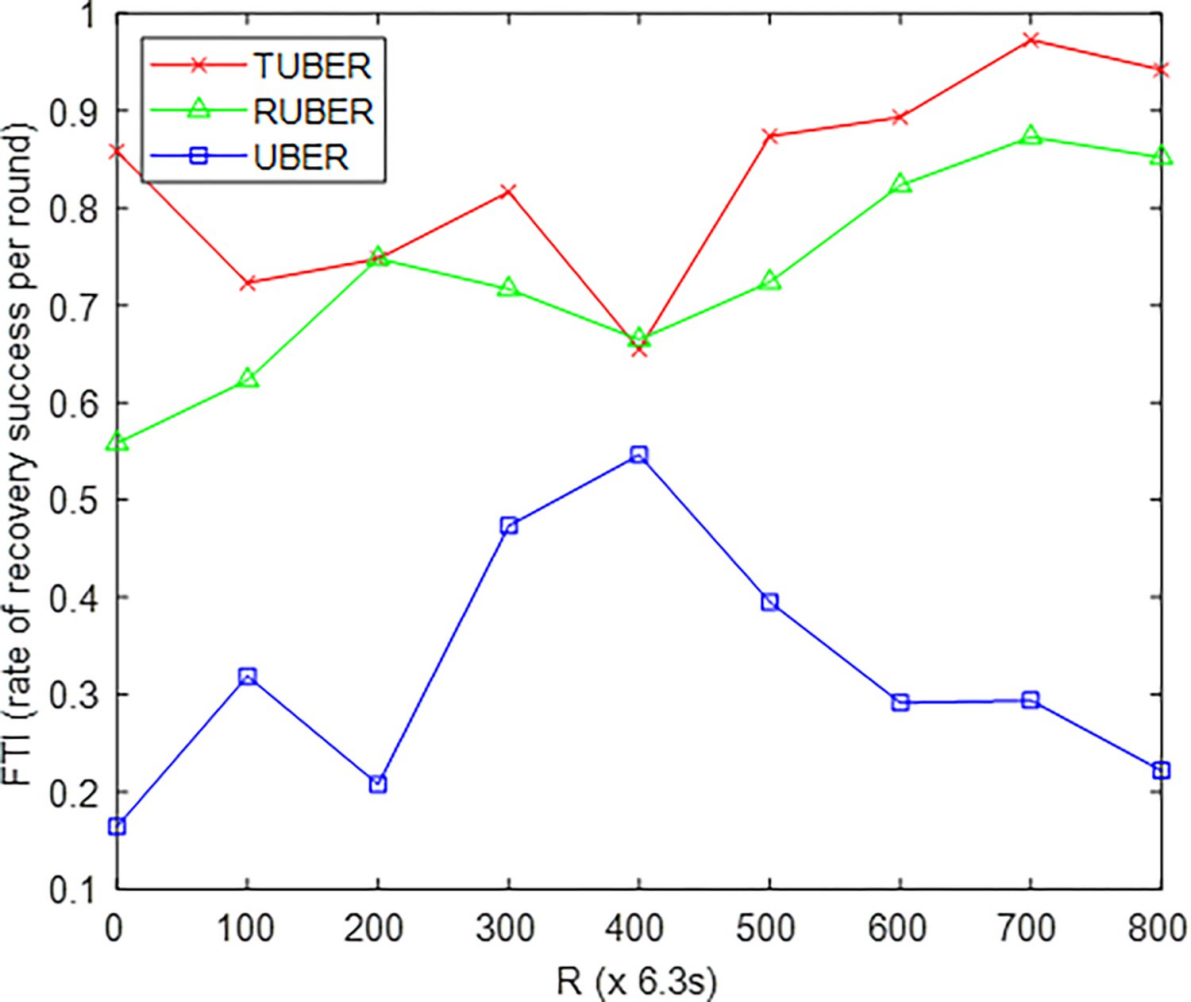
Fault tolerance index performance.

Fig 10 depicts the comparative plot of fault tolerance index (FTI) for TUBER with respect to RUBER and UBER. FTI is defined as the ratio of the number of successes recorded for faults recovered to the total number of aggregate fault outcomes (failures and successes) after *R* network operation rounds.

**Fig 11:**
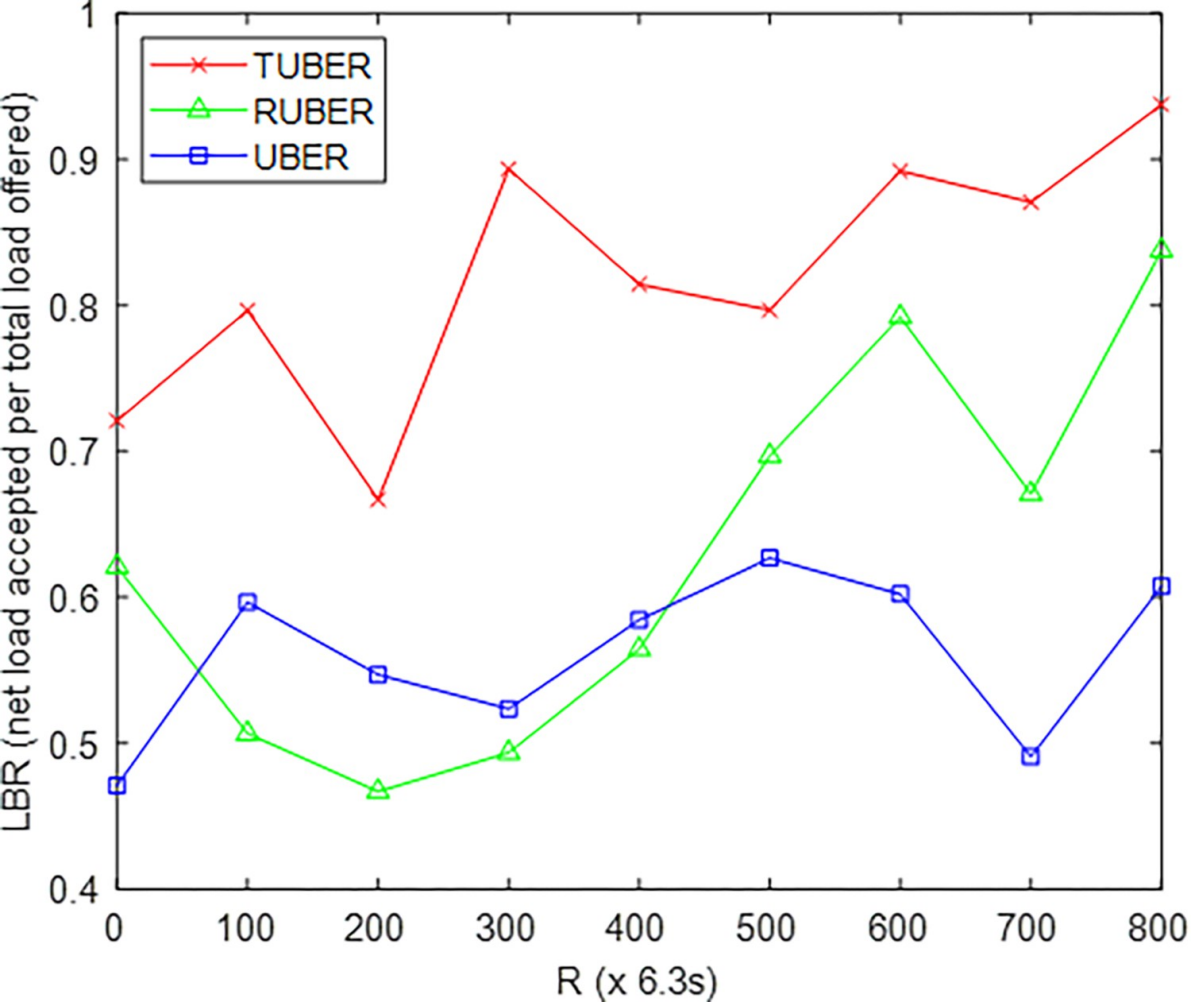
Load balancing ratio performance.

Fig 11 provides the comparative plot of load balancing ratio (LBR) for TUBER in comparison to RUBER and UBER. LBR is defined as the comparative ratio of net load successfully accepted by the MS to the total load offered by the HCLs, averaged for *R* network operation rounds.

**Fig 12:**
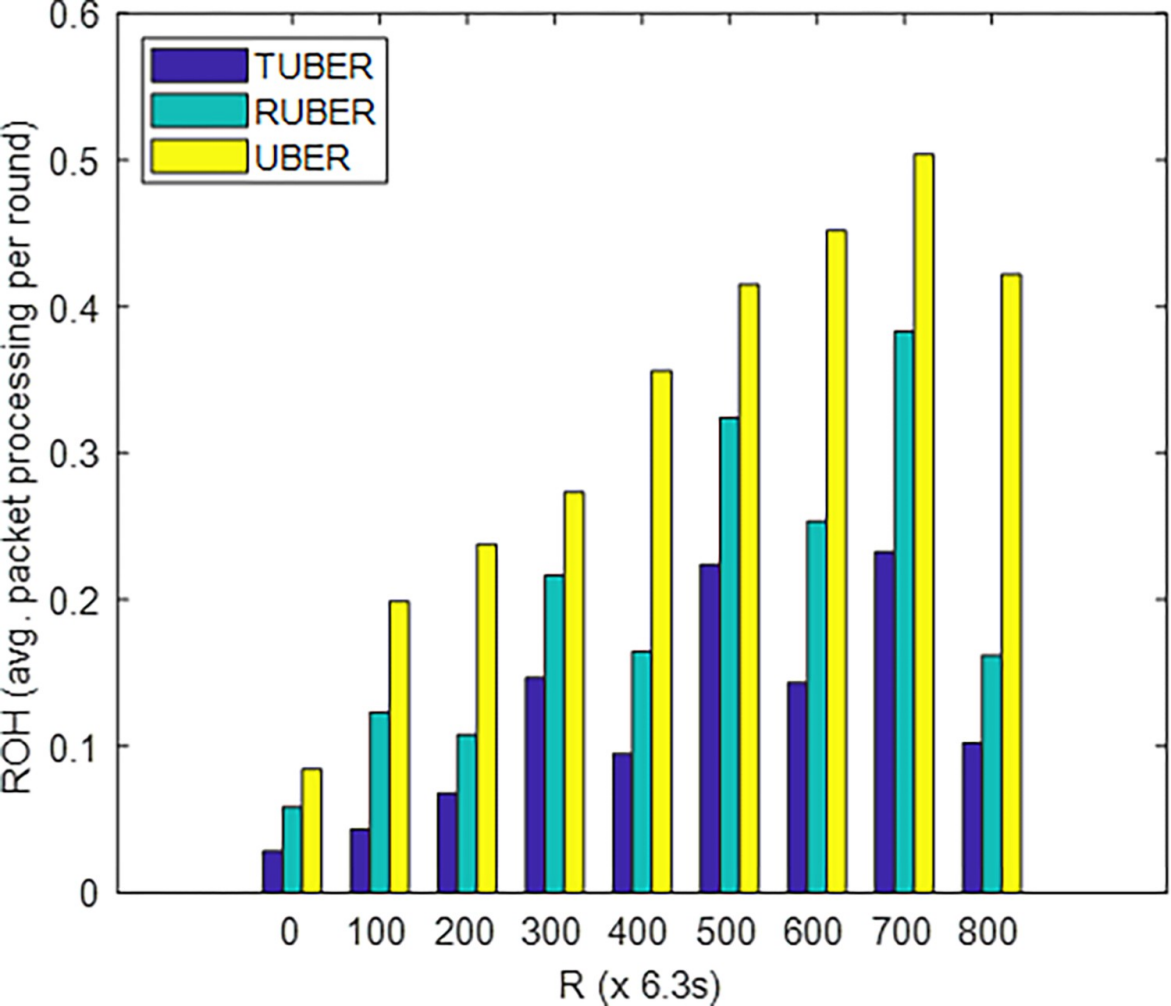
Routing overhead performance.

Fig 12 shows the comparative plot of routing overhead (ROH) for TUBER with respect to RUBER and UBER. ROH is defined as the ratio (in percentage) of packet processing (prior to actual data transmission) duration to network operation duration.

**Fig 13:**
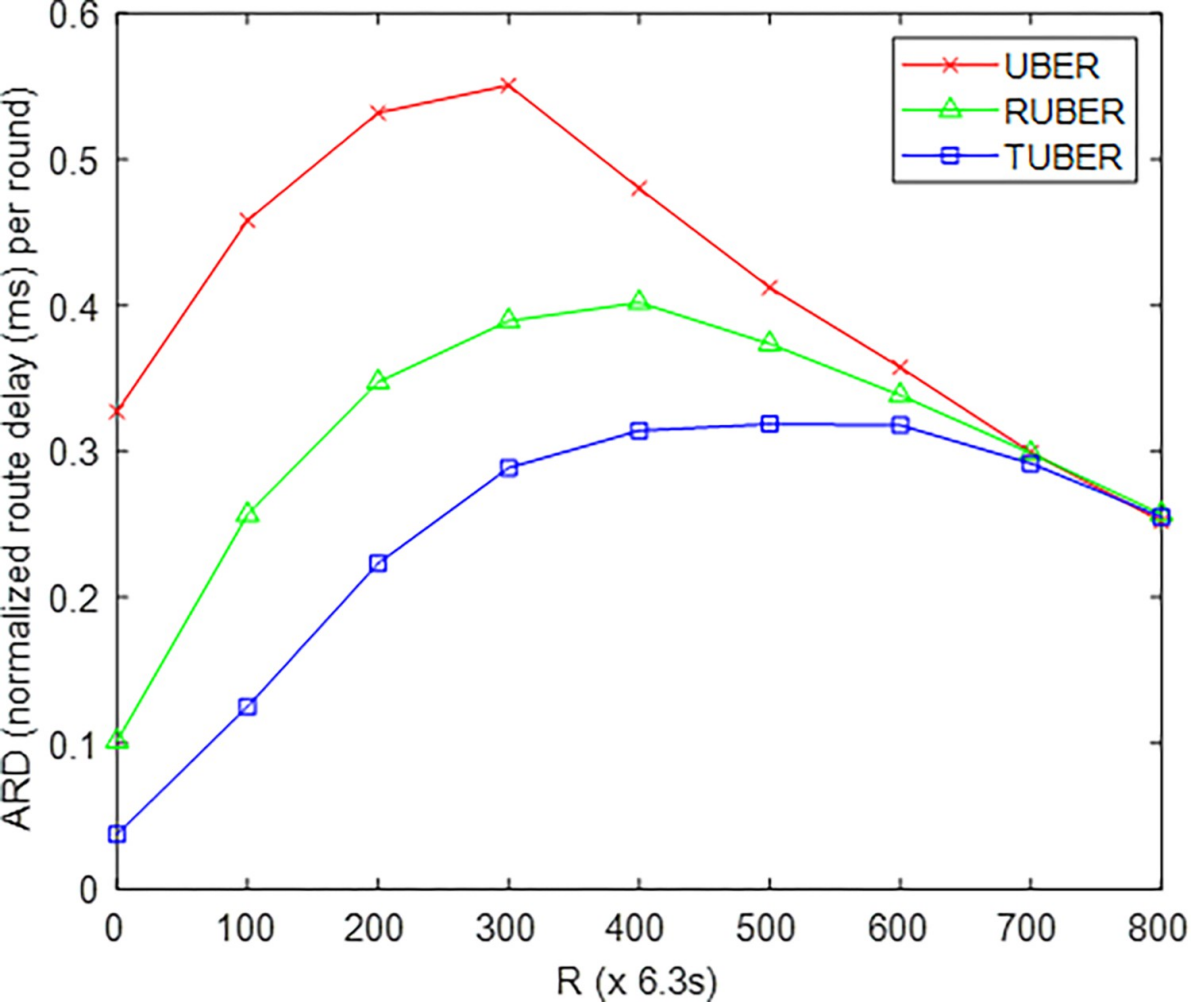
Average routing delay performance.

Fig 13 provides the comparative plot of average routing delay (ARD) for TUBER in comparison to RUBER and UBER. ARD is defined as the total duration measured from initial packet generation at the HCM to eventual delivery via the MS-to-LMS transmission link as a ratio of the total network duration.

**Fig 14:**
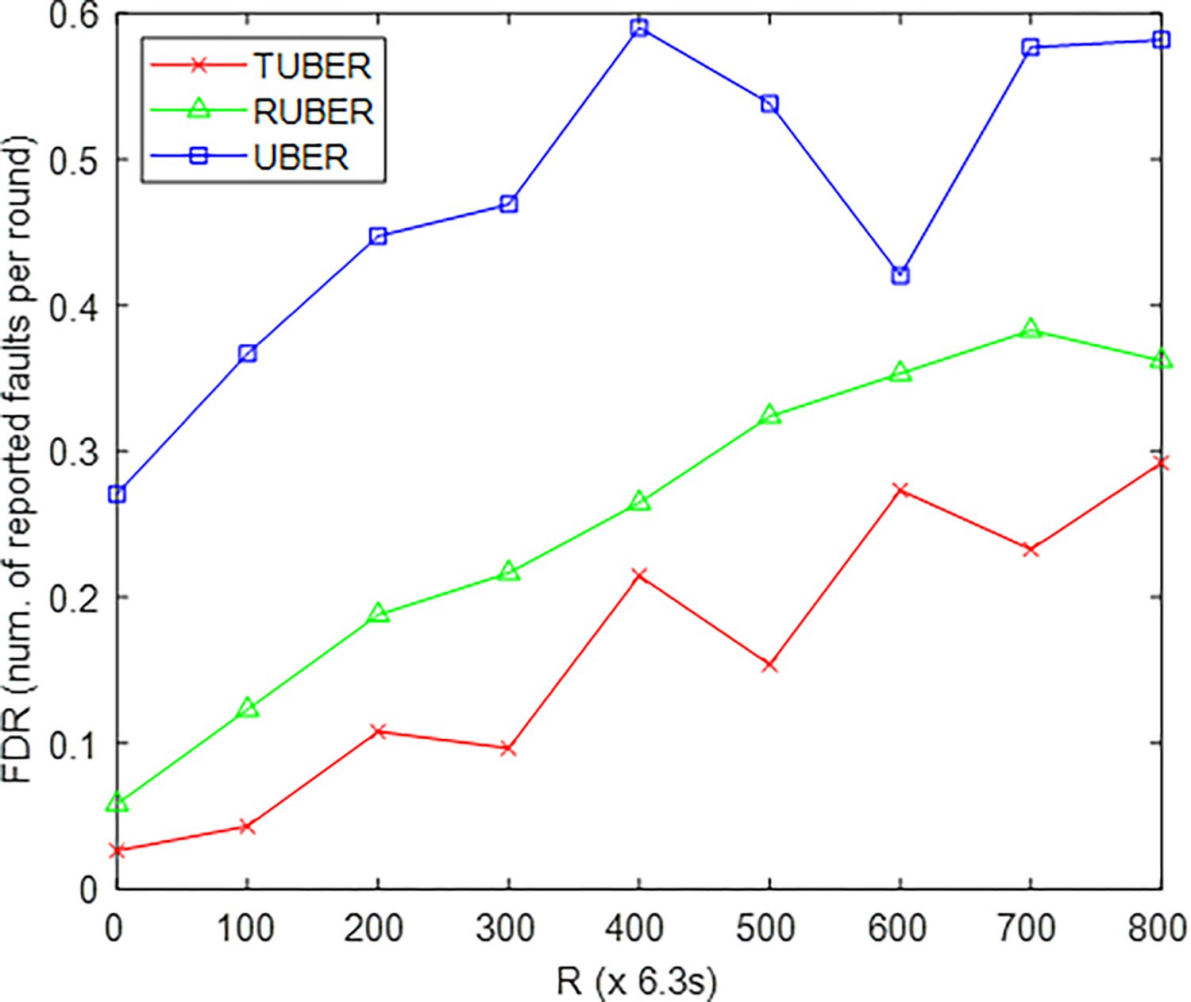
Failure detection ratio performance.

Fig 14 gives the comparative plot of failure detection ratio (FDR) for TUBER in comparison to RUBER and UBER. FDR is defined as the ratio of faults detected to the total number of faults occurrence at each specified round of network operation.

**Fig 15:**
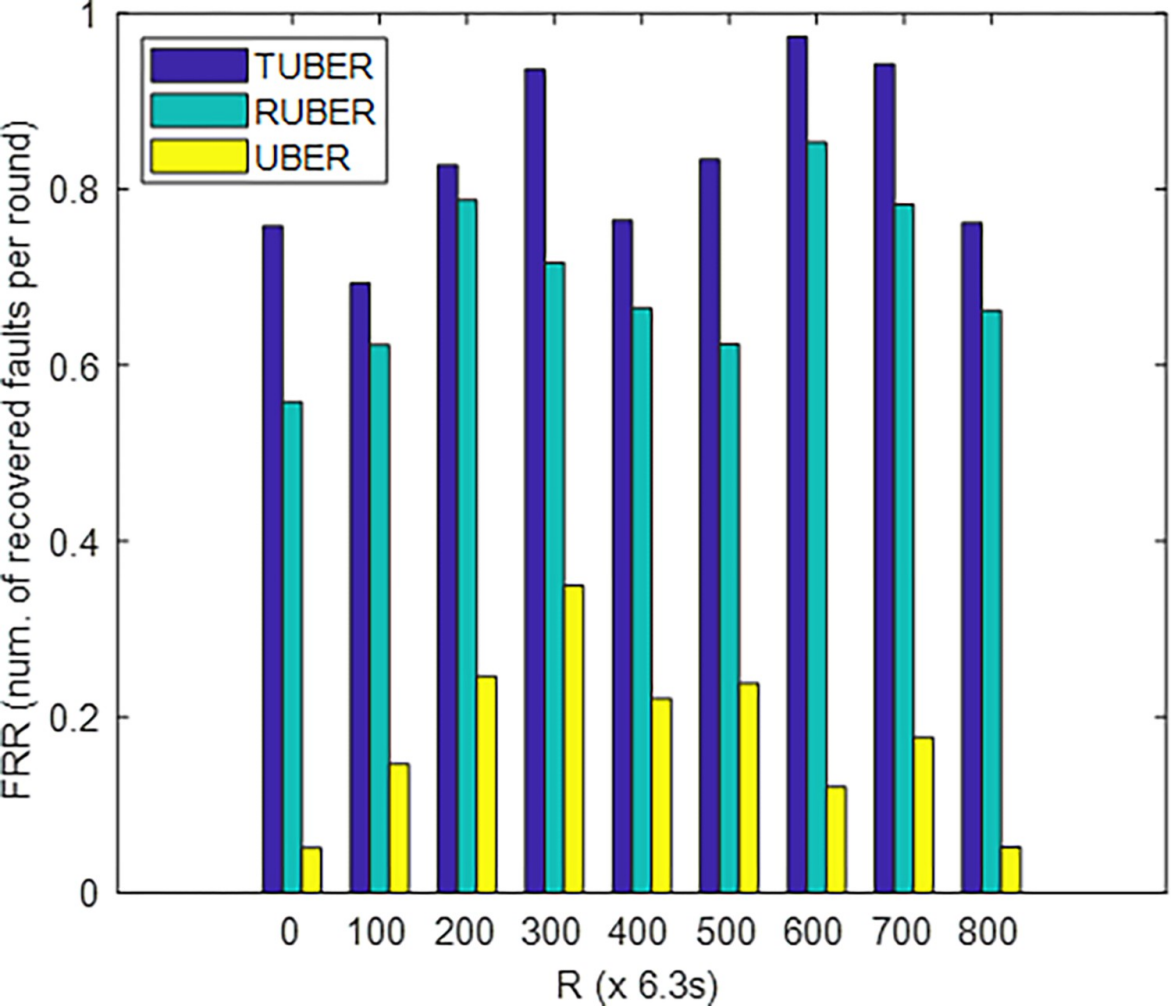
Failure recovery ratio performance.

Fig 15 depicts the comparative plot of failure recovery ratio (FRR) for TUBER in comparison to RUBER and UBER. FRR is defined as the ratio of faults recovered to the total number of faults occurrence at each specified round of network operation.

## 5. Discussions

### 5.1. Evaluation of cluster survival ratio (CSR) performance

From Fig 5, TUBER demonstrated higher cluster survival ratio with 21.04% and 36.25% improvements over RUBER and UBER, respectively. The reason for these performance gains are the redundancy minimization techniques (partial re-clustering, decentralized fault-tolerance, cluster survivability measures) and energy-efficient HCL-based joint handshaking approach which prolongs cluster lifespan by reducing energy tax on exchange of data and control packets.

### 5.2. Evaluation of network stability (NST) performance

From Fig 6, TUBER exhibited higher network stability by yielding improvements of 16.05% and 24.81% over RUBER and UBER, respectively. The justification for these performance gains is the synchronized clustering-with-backup process and load balancing strategy for curbing intra-cluster congestion, cluster (inter-cluster and HC-to-MS) overload, and cluster re-scaling/re-adjustment issues.

### 5.3. Evaluation of energy dissipation ratio (EDR) performance

From Fig 7, TUBER displayed lower energy dissipation ratio with gains of 21.82% and 34.53% over RUBER and UBER, respectively. These performance improvements are technically justified by the utilization of dynamic/linearized $E_{TAX}^{th}$ thresholding, implementing load balancing condition, and adoption of the more lifetime-sensitive ratio *E*_rsd_/*E*_cor_ for heterogeneous battery consumption trends in the synchronous clustering-with-backup process.

### 5.4. Evaluation of network coverage (COV) performance

From Fig 8, TUBER exhibited higher network coverage by recording improvements of 10.32% and 15.65% over RUBER and UBER, respectively. These performance gains are technically justified by the adoption of MS for network coverage extension, reliable HCL-to-MS transmission link expansion, and least-cost multi-hop backup data transmission chain construction.

### 5.5. Evaluation of packet delivery ratio (PDR) performance

From Fig 9, TUBER recorded higher packet delivery ratio by showing enhancements of 14.63% and 38.32% over RUBER and UBER, respectively. These performance enhancements are as a result of the rapid re-affiliation (low-cost partial re-clustering), effective fault repair, and minimum-hop neighborhood recovery procedures for minimizing cases of high retransmission rates, connectivity loss, and frequent packet drops.

### 5.6. Evaluation of fault tolerance index (FTI) performance

From Fig 10, TUBER exhibited higher fault tolerance index by recording gains of 12.03% and 61.07% over RUBER and UBER, respectively. The technical justification for these performance improvements is the decentralized fault-tolerant strategy (recovery is highly flexible, strategically tailored and adaptive to each SN) together with robust failure detection, rapid re-affiliation (low-cost partial re-clustering), and minimum-hop neighborhood recovery procedures.

### 5.7. Evaluation of load balancing ratio (LBR) performance

From Fig 11, TUBER displayed higher load balancing ratio by 23.54% and 31.66% improvements over RUBER and UBER, respectively. The major reason for these performance improvements is the adoption of the more lifetime-sensitive ratio *E*_rsd_/*E*_cor_ suitable for heterogeneous battery consumption trends in the synchronous clustering-with-backup process. Another justification for this is the decentralized and balanced fault-tolerant strategy (recovery is highly flexible, strategically tailored and adaptive to each SN).

### 5.8. Evaluation of routing overhead (ROH) performance

From Fig 12, TUBER demonstrated lesser routing overhead by 39.61% and 63.20% gains over RUBER and UBER, respectively. The justification for these enhancements is the simplified algorithmic (time and processing) execution costs, simplified HCL-based joint handshaking approach, rapid cluster re-affiliation, effective HCL-to-MS data transmission chain, and recovery route resolution/reconstruction.

### 5.9. Evaluation of average routing delay (ARD) performance

From Fig 13, TUBER displayed lower average routing delay with 27.28% and 68.96% improvements over RUBER and UBER, respectively. The justification for these performance gains are the delay minimization elements (simplified handshaking within and across clusters, synchronous clustering-with-backup processing, rapid backup route construction for inter-cluster transmissions, transmission window maintenance, retransmission limit implementation) of the adopted redundancy minimization mechanism.

### 5.10. Evaluation of failure detection ratio (FDR) performance

From Fig 14, TUBER exhibited lesser failure detection ratio by 36.61% and 66.19% improvements over RUBER and UBER, respectively. The technical reason for these improvements is that the simplified HCL-based joint handshaking, reduced control packets transmission, effective path resolution and prevention of cluster looping results in relatively less cases of network faults and improves the failure detection process.

### 5.11. Evaluation of failure recovery ratio (FRR) performance

From Fig 15, TUBER displayed higher failure recovery ratio by 16.28% and 78.63% improvements over RUBER and UBER, respectively. A key reason for these performance gains are the decentralized fault-tolerant strategy (recovery is highly flexible, strategically tailored and adaptive to each SN), rapid re-affiliation (low-cost partial re-clustering), effective fault repair, and minimum-hop neighborhood recovery procedures for successful recovery from SN and route failures.

Table 4 gives the summary of TUBER’s performance results in comparison with RUBER and UBER.

**Table 4:** Outline of TUBER’s performance comparison results.

Metric	RUBER	UBER
CSR	21.04%	36.25%
NST	16.05%	24.81%
EDR	21.82%	34.53%
COV	10.32%	15.65%
PDR	14.63%	38.32%
FTI	12.03%	61.07%
LBR	23.54%	31.66%
ROH	39.61%	63.20%
ARD	27.28%	68.96%
FDR	36.61%	66.19%
FRR	16.28%	78.63%

## 6. Conclusions

This paper addressed the time complexity and processing cost issues for smart wireless livestock sensor networks by developing a time-aware UAV-based energy-efficient reconfigurable routing scheme, referred to as TUBER. TUBER scheme employs a synchronized clustering-with-backup strategy, a minimum-hop neighborhood recovery mechanism, and a redundancy minimization technique. OMNET++ and MATLAB simulation experiments were carried out for comprehensive performance evaluation. Comparative network performance of the TUBER was investigated and evaluated with respect to the recoverable UAV-based energy-efficient reconfigurable routing (RUBER) and the UAV-based energy-efficient reconfigurable routing (UBER) schemes. With reference to best-obtained values, TUBER demonstrated marked performance gains in terms of cluster survival ratio, network stability, energy dissipation ratio, network coverage, packet delivery ratio, fault tolerance index, load balancing ratio, routing overhead, average routing delay, failure detection ratio, and failure recovery ratio. The obtained experimental results confirmed the relative effectiveness of TUBER against the two other compared routing schemes.

## 7. Future research works

Future directions in this project will focus on integrated UAV-WSN testbed development and deployment for experimental livestock monitoring at the campus of University of Hafr Al Batin. The purpose of embarking on this is to allow practical system evaluation and comparative assessment of actual field measurements with the recorded simulation results. Furthermore, critical stability issues (i.e., degradative effects of the increase in node density and the increase in LF network field) will be addressed in a forthcoming research paper by developing a low-cost scalable CBR scheme.

## Abbreviations and acronyms

Α: Path Loss Factor
ABC-PSO: Artificial Bee Colony Optimized Particle Swarm Optimization
AODV-SMS: Ad-hoc On-demand Distance Vector Routing for Single Mobile Sink
ARD: Average Routing Delay
AVG-STAT: Statistical Averaging Runs
BS: Base Station
CBR: Cluster-Based Routing
CC_x_: Clustering Costs
COV: Network Coverage
CSR: Cluster Survival Ratio
CTR_max_: Maximum Coverage
E_agg_: Collation Energy
E_cor_: Energy Consumption Rate
EDR: Energy Dissipation Ratio
E_EC_: Electronic Energy
E_idle_: Idling Energy
E_rsd_: Residual Energy
E_TAX_ TL: Energy Threshold Levels
E_tot_: Total Energy
FCGW: Fault-tolerant Clustering Routing Protocol based on Gaussian Network for Wireless Sensor Network
FDR: Failure Detection Ratio
FRR: Failure Recovery Ratio
FTI: Fault Tolerance Index
G_ADJ_: Adjacent Sensor Nodes
G_THCL_: Set of Neighboring Trial Herd Cluster Leads
HC: Herd Cluster
HCL: Herd Cluster Lead
HCL_PR_: Electability Probability
HCM: Herd Cluster Member
HYBRID: Hybrid Heterogeneous Routing Scheme
INET: Internet Link
IoT: Internet of Things
LBR: Load Balancing Ratio
LF: Livestock Farming
LFC: Livestock Farming Controller
LF-NS: Network Dimension
LIM_LOW_: Lower bound for HCL Contention
LIM_UP_: Upper bound for HCL Contention
LMS: Livestock Monitoring Server
MEU: Mobile End User
MS: Mobile Sink
MS-ALT: Altitude of Unmanned Aerial Vehicle
MS-DVCR: Mobile Sink using Directional Virtual Coordinate Routing
MS-SR: Beaconing Rate of Unmanned Aerial Vehicle
MS-TD: Tour Length of Unmanned Aerial Vehicle
MS-V: Speed of Unmanned Aerial Vehicle
NGW: Network Gateway
NST: Network Stability
PDR: Packet Delivery Ratio
PKS: Size of Data Packet
R: Specific Number of Network Operation Round
R_max_: Maximum operation round
ROH: Routing Overhead
RRM-WLDCNN: Resilient Routing Mechanism using Weisfeiler-Lehman kernel and Dual Convolutional Neural Network
RUBER: Recoverable Unmanned Aerial Vehicle-based Energy-efficient Reconfigurable Routing
SN: Sensor Node
SN-DEP: Deployed Sensor Nodes
SN-RS: Sensitivity of Receiver
TUBER: Time-aware Unmanned Aerial Vehicle-based Energy-efficient Reconfigurable Routing
UAV: Unmanned Aerial Vehicle
UBER: Unmanned Aerial Vehicle-based Energy-efficient Reconfigurable Routing
VBRP: Voronoi Based Rendezvous Points
WAP: Wireless Access Point
WSN: Wireless Sensor Network
ZGB: ZigBee Interface
Z_x,t_: Signal strength of Unmanned Aerial Vehicle Beacon
